# Commentary on: “The body social: an enactive approach to the self“. A tool for merging bodily and social self in immobile individuals

**DOI:** 10.3389/fpsyg.2015.00305

**Published:** 2015-03-19

**Authors:** Giulia Galli, Mariella Pazzaglia

**Affiliations:** ^1^IRCCS Santa Lucia FoundationRome, Italy; ^2^Department of Psychology, University of Rome “La Sapienza,”Rome, Italy

**Keywords:** embodiment, tool use, spinal cord injuries, bodily self, social self

The article by Miriam Kyselo titled *“The body social: an enactive approach to the self”* (Kyselo, 2014) raises a crucial issue and discusses how bodily and social aspects may merge together in a unique account of the human self. The author rightly suggests that we do not have to choose between the bodily and social dimensions of self.

The integration process between bodily and social self may fail in patients with a number of neurological and psychiatric diseases, as well as during certain experimental conditions (Lucci and Pazzaglia, 2015). Various neurological conditions produce disturbed self perceptions, for example disownership of one's hand in somatoparaphrenia (Vallar and Ronchi, 2009; Romano et al., 2014), desire for amputation in body integrity identity disorder (Sedda, 2011), unintended movements in alien hand syndrome (Della Sala et al., 1991), ownership for supernumerary limbs (Guterstam et al., 2011) or seeing own body from a third person perspective in out of body experiences (Blanke and Arzy, 2005). The illusory alterations of awareness of the bodily self can be also induced experimentally during the so-called “rubber hand illusion”—RHI (Botvinick and Cohen, 1998), where the subjective perceptual experience interfere with bodily self and the external bodily-shaped object is treated as part of the body. These clinical populations and experimental condition may challenge Kyselo's view, as bodily self could be disrupted despite a normal social self.

However, an example of the complementarity of bodily and social self are patients with spinal cord injury (SCI). “The loss of self is inherent in the social isolation of paralytics, who have furthermore become separated from their bodies by neural damage, and from their former identities by society” (Murphy, 2001; p. 227). SCI can leave a part of the body insensible and immobile, leading to specific disorders in the mental representation of one's own body and the sense of bodily self (Lenggenhager et al., 2012, 2013). After SCI, patients see their former life slip away and become restricted, and the social implications can be considerable. They must adapt to their new body and make significant changes in the way they function in the world. Here we propose that a medium that accepts self-multiplicity while also offering an integrative perspective on the self is possible. We consider how an assistive tool can change the manner in which people with massive disconnection between body and brain access to social world.

For people who chose to integrate their disability into their full sense of self, the wheelchair represents a changing paradigm, as highlighted by significant theoretical models (Papadimitriou, 2008; Standal, 2011) and experimental studies (Arnhoff and Mehl, 1963; Winance, 2006a,b; Higuchi et al., 2009; Olsson, 2012; Fuentes et al., 2013; Pazzaglia et al., 2013). When patients do not perceive the loss or limitation and instead focus on the power and joy of seeing the world, “*I could whiz around and feel the wind in my face again. Just being out on the street was exhilarating”* (Austin, 2014), the wheelchair becomes a symbol of both physical and social independence for the body injured. Even for people with life-threatening illnesses, this tool provides a way to be out in the world, continuing their lives and accessing the many realms of human experience (Karp, 2008).

The plastic shaping of the bodily self has already been demonstrated by multiple neuropsychological studies showing that tools can be integrated into the representation of one's own body (Maravita and Iriki, 2004; Cardinali et al., 2009; Tsakiris, 2010; Longo and Serino, 2012). In the process, known as embodiment, the tool is processed in the same way as a part of one's body (De Vignemont, 2011), allowing a new connection between the sense of having a modified functional body and the sense of self. In the case of SCI, the wheelchair, which contributes to the individual's conscious movement, can be added to the dynamic representation of patients' bodily self. From a physical perspective, the corporeal awareness of a tool emerges as the functional self with its new rules and novel ways of interacting with the world (Pazzaglia et al., 2013). Through the use of the wheelchair, the perceived bodily self is that of being functionally whole, enabling the immobile user to act in the world again.

The wheelchair becomes a vehicle of freedom of mobility and independence for newly abled adults, reconstructing their sense of self (Papadimitriou, 2008). A deep embodiment process of the en-wheeled body can occur, enhanced for both the loss of sensorimotor function (Pazzaglia et al., 2013) and the critical sense of protection and monitoring (Rossetti et al., 2015).

From a social perspective, the injury affects consciousness as it introduces a novel pervasive identity. The tool incorporation process described above involves new ways of being in the world, i.e., the relations between self and others and the world (Winance, 2006a,b; Papadimitriou, 2008). The wheelchair increases permeability of the boundary between disabled and non-disabled individuals, directly mitigating the impairment and thereby helping the user escaping disability (Nario-Redmond et al., 2013). Functional and smooth wheelchair use significantly contributes to the production of a new identity and allows the individual to cope with his disability by emphasizing one's strengths, interests, and efficacy. It has the liberating aspect of providing opportunities to participate in social activities, through the new form of the body. In this case, disablement is not simply a physical affair; rather, it is a lifelong process of adjusting to changed circumstances (Pentland et al., 2002) that ultimately results in a transformation from disabled to newly abled. Being active and engaged constitutes a starting point for restructuring the social self. Emerging empirical data support the importance of the social context in shaping self-perceptions after a SCI (Fuhrer, 1996). Several surveys of patients with SCI revealed that their self-reported quality of life was only slightly different from that of control subjects without SCI (Stensman, 1994). Moreover, the quality of life for this population, regardless of severity of impairment, consisted of various themes (Manns and Chad, 2001) such as accessibility, emotional well-being, stigma, spontaneity, relationships and social function, occupation, finances and independence, additionally to the expected physical variables such as functionality and physical well-being. Similarly, patients with locked-in syndrome can adjust very well to the objective physical change and actually feel the same as before (Nizzi et al., 2012), although changes in their self-awareness and quality of life are not mediated by any functional prosthetic tool, which in this case would not have the meaning of unifying bodily and activity extremely restricted of social self.

The effort to understand how people maintain psychological well-being despite physical and social challenges is a core topic of many human sciences. The field has shifted from a medical model of disability as a functional limitation of living without movement and sensation, to a social model that includes consequent relational problems (Altman, 2001; Williams, 2001; Cole, 2004; Darling and Heckert, 2010). Both physical and emotional adjustments that follow wheelchair use could result in a new bodily and social-self representation, in which the person may incorporate the wheelchair (Fuentes et al., 2013; Pazzaglia et al., 2013). Bodily changes can affect and be integrated into our self as a disordered organism regains a state of order (Goldstein, 1939). The fact that quality of life often equates with social rather than physical status (Gosseries et al., 2009) makes sense when the boundaries of self are not determined by bodily processes alone, but are instead plastic in terms of relational and social interactions. Although the relationship between actual bodily states and embodied cognition has been widely explored, little is known about how changes in the connection between brain and body periphery, occurring after a traumatic SCI, influence higher-level embodied self such as perspective-taking, inter-subjectivity and group membership, which makes these topics extremely challenging for future investigations.

As an individual has to change adaptively (Thompson, 2007) by flexibly regulating his own identity (Di Paolo, 2005), it is likely that when discussing individuality, SCI patients do not view their self as primarily physical or social, but rather including both aspects of self-definition and the embodiment of the functional tool that permits them to re-negotiate their self as a whole.

## Conflict of interest statement

The authors declare that the research was conducted in the absence of any commercial or financial relationships that could be construed as a potential conflict of interest.
